# MEK is a promising target in the basal subtype of bladder cancer

**DOI:** 10.18632/oncotarget.27767

**Published:** 2020-11-03

**Authors:** Nathan M. Merrill, Nathalie M. Vandecan, Kathleen C. Day, Phillip L. Palmbos, Mark L. Day, Aaron M. Udager, Sofia D. Merajver, Matthew B. Soellner

**Affiliations:** ^1^Department of Internal Medicine, University of Michigan, Ann Arbor, MI, USA; ^2^University of Michigan Rogel Cancer Center, Ann Arbor, MI, USA; ^3^Department of Chemistry, University of Michigan, Ann Arbor, MI, USA; ^4^Michigan Center for Translational Pathology, University of Michigan, Ann Arbor, MI, USA; ^5^Department of Urology, University of Michigan, Ann Arbor, MI, USA

**Keywords:** bladder cancer, drug screen, 3D culture, basal bladder cancer, MEK inhibition

## Abstract

While many resources exist for the drug screening of bladder cancer cell lines in 2D culture, it is widely recognized that screening in 3D culture is more representative of *in vivo* response. Importantly, signaling changes between 2D and 3D culture can result in changes to drug response. To address the need for 3D drug screening of bladder cancer cell lines, we screened 17 bladder cancer cell lines using a library of 652 investigational small-molecules and 3 clinically relevant drug combinations in 3D cell culture. Our goal was to identify compounds and classes of compounds with efficacy in bladder cancer. Utilizing established genomic and transcriptomic data for these bladder cancer cell lines, we correlated the genomic molecular parameters with drug response, to identify potentially novel groups of tumors that are vulnerable to specific drugs or classes of drugs. Importantly, we demonstrate that MEK inhibitors are a promising targeted therapy for the basal subtype of bladder cancer, and our data indicate that drug screening of 3D cultures provides an important resource for hypothesis generation.

## INTRODUCTION

Bladder cancer is the most frequent cancer of the urinary system in the United States with nearly 82,000 new cases each year and 18,000 deaths, affecting men more often, in a 3:1 ratio [1]. Bladder cancer can be divided broadly into non-muscle invasive bladder cancer (NMIBC) and muscle invasive bladder cancer (MIBC). MIBC can be further sub-divided at the molecular level by the expression of RNA biomarkers between classes, that define basal and luminal characteristics [2–8]. The standard of care for intermediate- to high-risk NMIBC has been Bacille Calmette-Guerin (BCG) since its introduction in 1976, with cystectomy as the recommended standard of care in refractory, high risk disease [9, 10]. While NMIBC makes up 70–80% of total cases, tumor recurrence is frequent and ~30% of cases progress to MIBC [11]. Neoadjuvant chemotherapy prior to radical cystectomy (RC) for MIBC is the standard of care, though the absolute survival benefit is small, and some patients progress during chemotherapy [12]. While progress has been made in the prediction of sensitivity to platinum-based chemotherapies [13, 14], identifying targeted therapies specific to each patient remains a critical need for those patients who progress during chemotherapy and/or after cystectomy.

There have been several large-scale screening efforts in bladder cancer cell lines using 2D cultures. The Broad Institute Cancer Cell Line Encyclopedia (CCLE) has characterized 56 urinary tract carcinomas and screened many of these cell lines against 24 drugs [15, 16]. The Genomics of Drug Sensitivity in Cancer (GDSC) represents one of the largest efforts in total drugs, screening 19 bladder cancer cell lines against 518 drugs [16, 17]. Additional efforts to identify therapeutic targets in bladder cancer include CRISPR screening and epigenetic approaches [18–20]. However, we now know that screening in 3D culture is superior to 2D culture, with improved *in vivo* relevance [21–26]. Indeed, screening in 3D using ultra-low attachment plates is ideal for bladder cancer cell culture [27], and this method has been utilized in seminal studies for screening patient-derived organoids (PDOs) to predict patient response to drug treatments [28, 29]. While direct screening of patient material is cutting edge and most representative of drug response for that particular patient, such material is typically very limited, which restricts the size of a potential drug screening library. Additionally, bladder cancer cell lines have undergone comprehensive molecular profiling allowing rapid correlational pairing of molecular profile with 3D phenotype [7]. Therefore, there is utility in screening bladder cancer cell lines in large drug screens in 3D cultures to identify novel therapeutic options for future testing in PDOs and, ultimately, patients.

In this work, we treated 17 established bladder cancer cell lines with 652 investigational small-molecules and 3 clinically relevant combinations in 3D cell culture. From this screening, we identified compounds and classes of drugs with promising efficacy in bladder cancer. Then, utilizing established genomic and transcriptomic data for these bladder cancer cell lines, including prioritized mutations, copy number variants, and RNA-based molecular subtyping [7, 15], we correlated these molecular parameters with drug response to identify potentially novel groups of tumors that are vulnerable to specific drugs or classes of drugs. Importantly, we showed that MEK inhibitors are a promising targeted therapy in basal subtype bladder cancer cell lines, and our data indicate that drug screening of 3D cultures provides an important resource for future hypothesis generation.

## RESULTS

### 3D drug screen in bladder cancer cell lines

**Figure 1:**
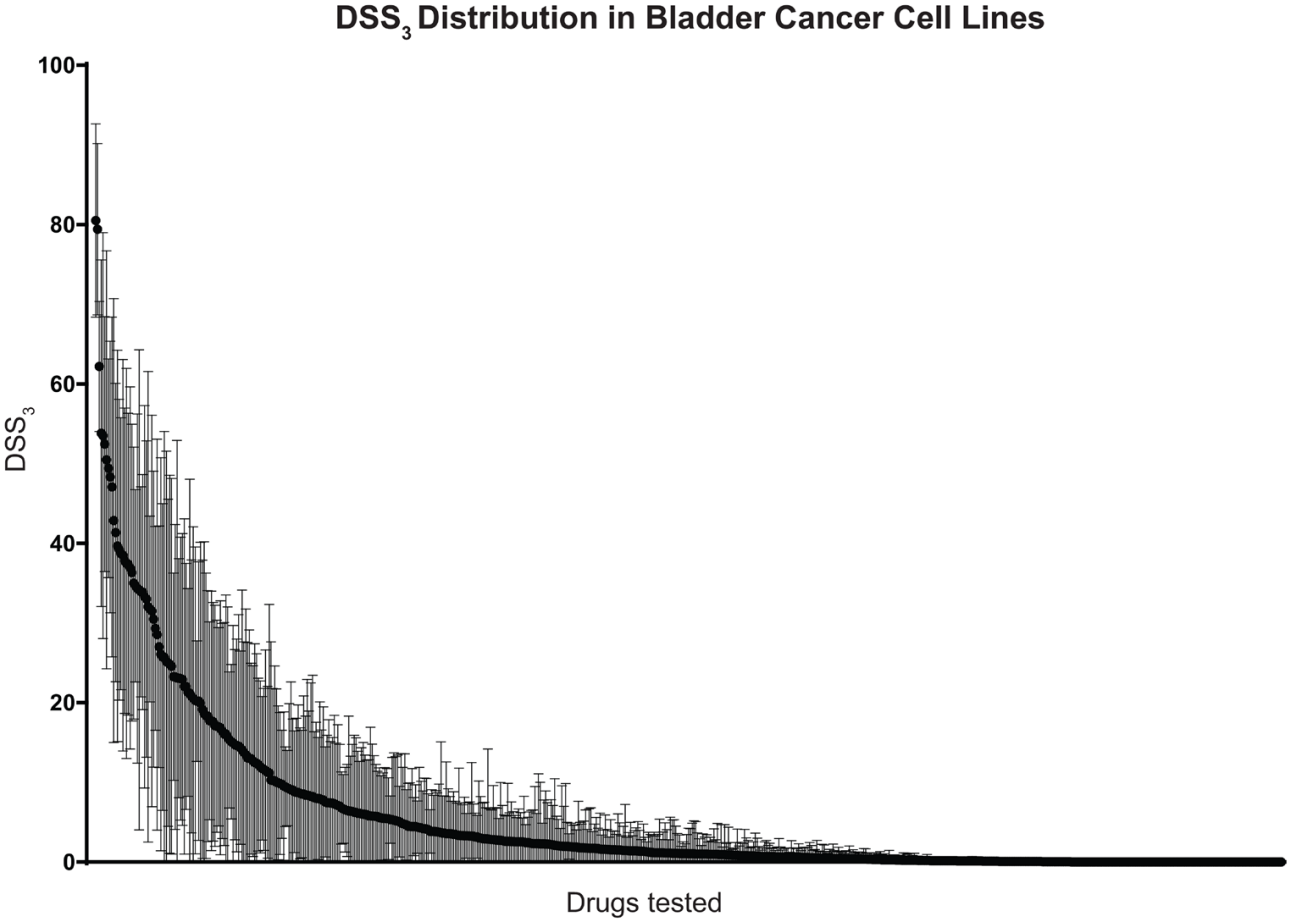
Distribution of drug sensitivities across bladder cancer cell lines. 652 investigational drugs and 3 clinically relevant combinations were tested against 17 bladder cancer cell lines in 3D cell culture. Drugs are ordered along x-axis by average drug sensitivity (DSS_3_), starting with the most sensitive drug. Black circles indicate average DSS_3_ and brackets indicate standard deviation across the 17 cell lines.

To examine bladder cell line drug sensitivity, we screened 17 cell lines against 652 investigational small-molecules and 3 clinically relevant combinations in 3D cell culture. From this drug sensitivity data, we calculated a drug sensitivity score 3 (DSS_3_) for each compound, an advanced drug sensitivity metric that uses the IC50, maximum inhibition, and drug concentration range to score drug sensitivity from 0 (no effect) to 100 (complete effect), Supplementary [30]. We plotted the average and standard deviation for each drug across the 17 cell lines to visualize the DSS_3_ spread in data, Figure 1. Scores of > 59 are considered “very active”, 30–59 “active”, 21–29 “semi-active”, 9–20 “low active”, and < 9 “inactive” [30]. From our drug screening, we identify 3 drugs (0.5%) as very active, 30 drugs (4.6%) as active, 20 drugs (3.0%) as semi active, 56 drugs (8.5%) as low active, and the remaining 547 (83.4%) as inactive (Supplementary).

We identify romidepsin, bortezomib, and triptolide as “very active” compounds across the 17 bladder cancer cell lines, on the basis of their DSS_3_. Romidepsin is a histone deacetylase (HDAC) inhibitor with an average DSS_3_ of 80.5 and a standard deviation of 12.1. HDAC inhibitors have been reported previously as potential therapeutic in bladder cancer and our results identify romidepsin and panobinostat (an “active: compound) as active pan-HDAC inhibitors. Bortezomib is a proteasome inhibitor with an average DSS_3_ of 79.4 and a standard deviation of 10.8. Proteasome inhibitors have been reported as potential therapeutics based on promising pre-clinical data and we identify bortezomib and delanzomib (an “active” compound) as potent proteasome inhibitors. Triptolide is an inhibitor of RNA polymerase I and II–dependent transcription with an average DSS_3_ of 62.2 and a standard deviation of 8.2. The 30 “active” compounds include both chemotherapeutics and targeted agents, many of which are currently utilized in the treatment of bladder cancer, such as gemcitabine, paclitaxel, vinblastine, and doxorubicin.

We screened three therapeutically relevant combinations with the top dose as the Cmax of each compound, serially diluted 1:5 to generate a dose response curve. For the combination of methotrexate, vinblastine, doxorubicin, and cisplatin (MVAC), a standard of care therapy in first-line therapy in MIBC, we observe a large spread in response, with a standard deviation in the DSS_3_ of 20.2. Four cell lines have a DSS_3_ as “active” or “very active” with most responses however, in the range of low active (8/17 cell lines). The overall average DSS_3_ for MVAC is 19.9. Because the top dose of the combinations is the Cmax, a better measure of therapeutic response for these combinations is the maximum response, which ranges from 52% to 100% in these cell lines (Supplementary), with an average of 81.1. The cisplatin and gemcitabine, alternative first line or second line combination, has a similar spread in data (DSS_3_ standard deviation = 15.2), but lower DSS_3_ values (average DSS_3_ = 8.2), and a wider spread in maximum response (0% to 100%, average = 65.0). Carboplatin and paclitaxel, another common first-line therapy, had the largest average DSS_3_ value and spread in data (average = 36.9, standard deviation = 22.7), but the lowest average maximum response of 53.9.

### Genomic correlates of drug sensitivity in 3D bladder cancer cell line cultures

**Figure 2:**
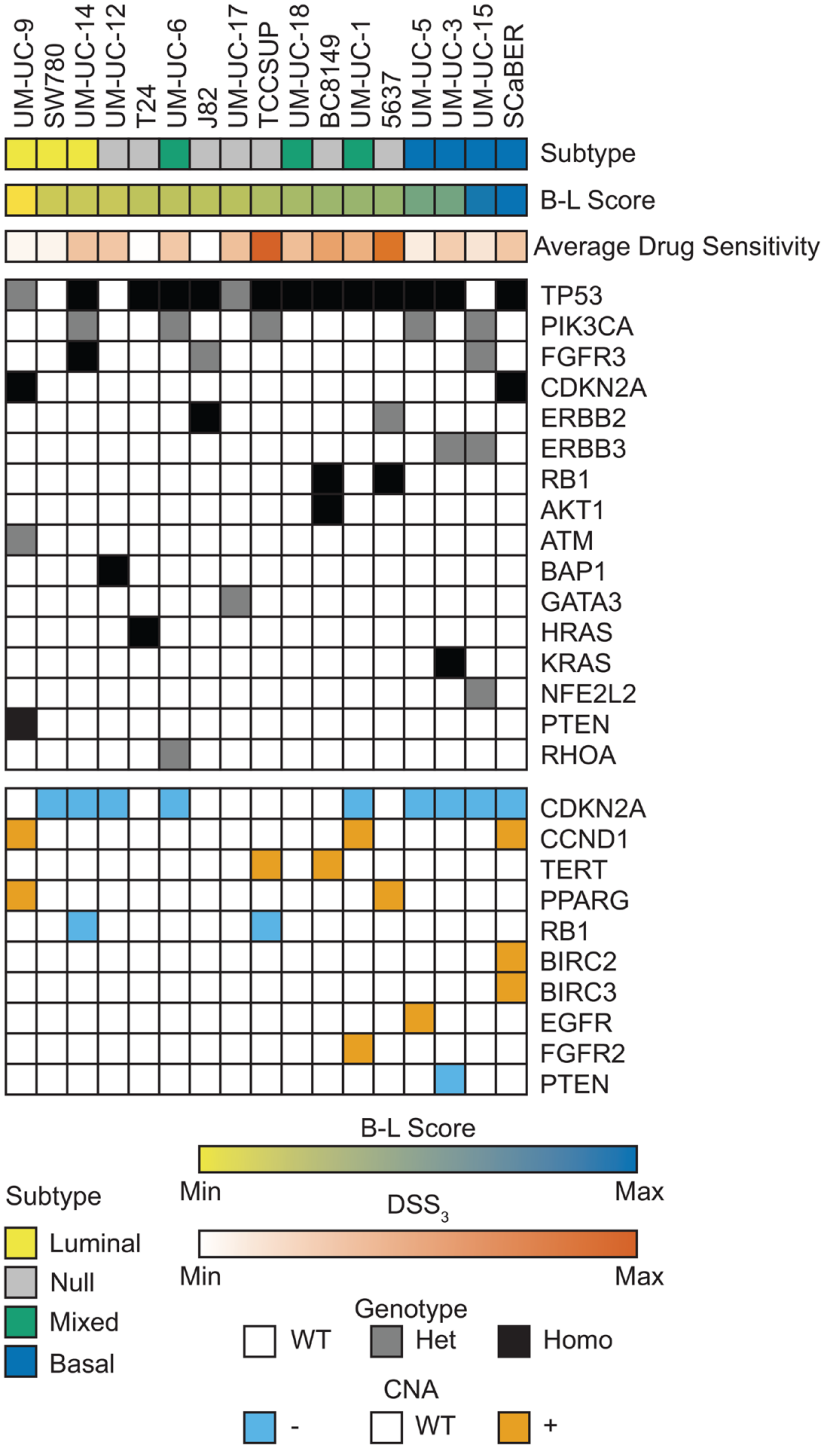
Genomic landscape of bladder cell lines. Bladder cancer subtype is indicated across the top row with each column representing an individual cell line. B-L Score was calculated from normalized RNA sequencing and is shown as a gradient from luminal (yellow) to basal (blue). Average drug sensitivity shows the average DSS_3_ across all drugs for each cell line. Mutation and genotype are indicated for all prioritized mutations present in 1+ cell line. CNA are indicated for high level amplifications (≥ 2 copy gain) and deep deletions (≥ 2 copy loss) in 1+ cell line.

We utilized DNA and RNA sequencing data previously published by our group, and the CCLE to examine the impact of molecular characteristics on drug sensitivity in these cell lines (see Materials and Methods for details), Figure 2 [7, 15]. As expected, the most prevalent somatic alteration in these bladder cancer cell lines was inactivating TP53 mutations, which were present in 14/17 cell lines, typically accompanied by loss of heterozygosity (LOH). We found that TP53 homozygous-mutant cell lines were more sensitive to onalespib (an Hsp90 inhibitor) and clofarabine (a purine nucleoside anti-metabolite) than TP53 wild-type (WT) cells, Supplementary Figure 1. The next most frequent mutations were oncogenic activating PIK3CA and FGFR3 mutations, which were present in 5/17 and 3/17 cell lines, respectively. We sought to identify drugs where there was a significant difference in DSS_3_ between groups (mutant and WT) and at least one group had a DSS_3_ > 10, identifying only AZD-8186 as more effective in PIK3CA WT cell lines, Supplementary Figure 2. If we include PTEN deletion with the PI3K group, because PTEN negatively regulates PI3K, we lose the correlation with AZD-8186 and instead observe a single robust correlate for MLN2238 (a proteasome inhibitor), Supplementary Figure 3. FGFR inhibitor response was not correlated with FGFR3 mutation status for any of the FGFR inhibitors in the panel (erdafitinib, AZD4547, BGJ398, and CH5183284). There were an additional 13 mutations present in 1-2 cell lines, including inactivating CDKN2A, RB1, and PTEN mutations with LOH and activating oncogenic ERBB2, AKT1, HRAS, and KRAS mutations. We did not observe increased ERBB2 inhibitor (lapatinib, canertinib, sapatinib, mubritinib, GW2580, dacomitinib, and WZ4002) sensitivity in ERBB2 activating mutant cell lines, in contrast to similar studies using 2D cell culture [31]. The AKT1 mutated PDX cell line (BC8149) is sensitive to many PI3K/AKT/mTOR inhibitors, while HRAS and KRAS mutated lines (T-24 and UM-UC-3) are largely insensitive to most PI3K/AKT/mTOR inhibitors.

We integrated the somatic mutation and CNA status to generate mutant groups for comparison. Combining CDKN2A loss-of-function mutants with CDKN2A CNA deep-deletion, we observe significant correlations with CDKN2A loss and poor response to cladribine or clofarabine, both purine analogs, as well as panobinostat and mocetinostat, both HDAC inhibitors, Supplementary Figure 4. If we combine RB1 loss-of-function mutants with RB1 CNA deep-deletion, we observe a significant correlation with RB1 loss and average drug sensitivity, where cells with RB1 loss respond more favorably to targeted agents and chemotherapeutics, Supplementary Figure 5.

### MEK inhibition correlates with basal subtype in bladder cancer cell lines

**Figure 3:**
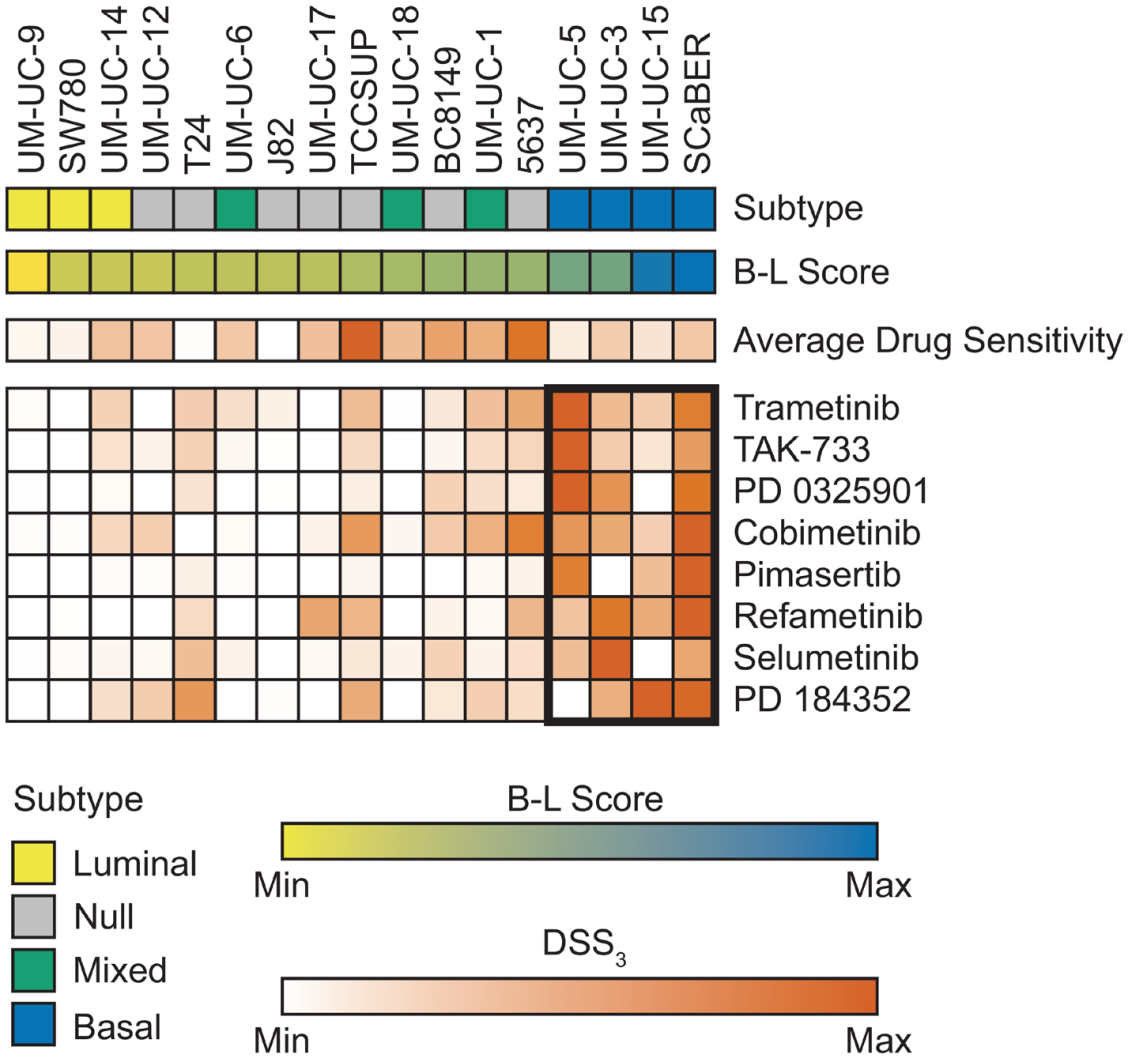
MEK inhibitors show strongest response in basal bladder cell lines. Bladder cancer subtype is indicated across the top row with each column representing an individual cell line. B-L Score was calculated from normalized RNA sequencing and is shown as a gradient from luminal (yellow) to basal (blue). Average drug sensitivity shows the average DSS_3_ for each cell line. Average DSS_3_ of MEK inhibitors for each cell line shown with average DSS_3_ > 0. MEK inhibitors ordered by average basal response with the best response at the top. Basal cell lines marked with bolded rectangle.

**Figure 4:**
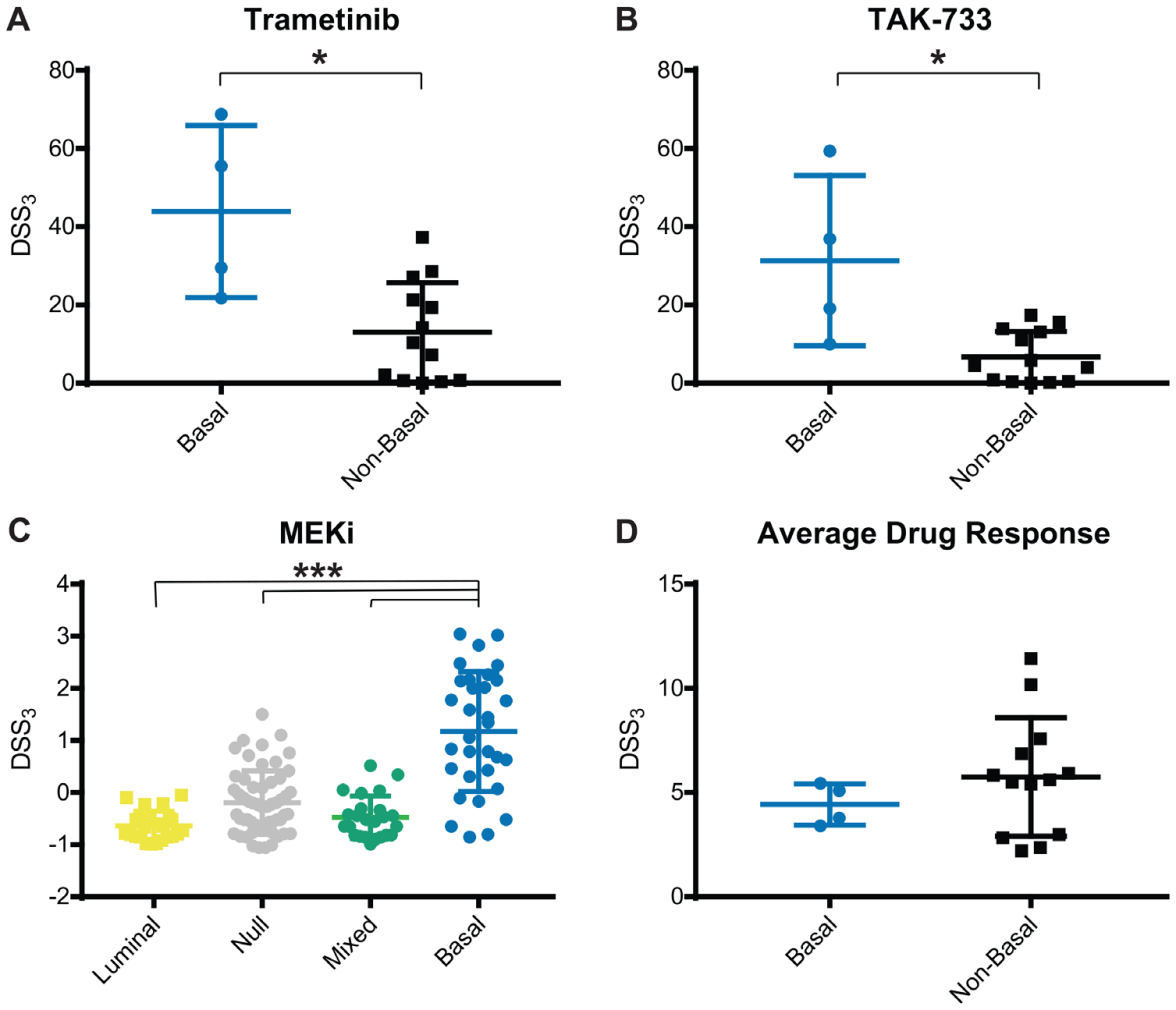
MEK inhibitor response correlates with basal subtype. Average and standard deviation for DSS_3_ response to (**A**) Trametinib, (**B**) TAK-733, (**C**) Normalized MEK inhibitors, and (**D**) Average drug response, grouped by cell line subtype. Each point represents an individual cell line. Center line is average and brackets are standard deviation. Significance determined using Mann-Whitney test, ^*^ *p* < 0.05, or Kruskal-Wallis with Dunn test for multiple comparisons, ^***^ *p* < 0.001.

We next wanted to determine if bladder cancer subtype correlated with drug response. Cell lines were classified as luminal, basal, null, or mixed based on RNA sequencing and B-L scoring [7, 32] (see Materials and Methods for details). We observed a strong correlation with MEK response in basal bladder cancer, Figure 3. Across 8 MEK inhibitors, we observed this same trend, with 2 very active inhibitors, 2 semi-active inhibitors, 3 low active inhibitors, and 1 inactive, but with a measurable response. The very active MEK inhibitors, Trametinib and TAK-773, both had a significantly higher MEK DSS_3_ in basal cell lines vs. other subtype, Figure 4A and 4B. When we normalized the response for all 8 MEK inhibitors, we saw both a significant difference in MEK response in basal vs. other (all remaining subtypes clustered, as well as basal vs. each other subtype, Figure 4C. Average drug response does not correlate with bladder subtype, Figure 4D. In agreement with this finding, we tested another basal bladder cancer cell line, UM-UC-13, and observed excellent anti-proliferative activity with MEK inhibitors (Supplementary Table 1). The only other drug response that we found to correlate with subtype was atuveciclib, a PTEFb/CDK9 inhibitor, with the basal subtype, Supplementary Figure 6.

## DISCUSSION

There is an increasing amount of literature that shows growing cells in 3D culture offers advantages over 2D, most importantly in that 3D cell culture signaling is more faithful of *in vivo* signaling [21–26]. This distinction is critical because we found many key drugs have differential responses when comparing 2D and 3D screening results, even when all other components of the assay are identical (Supplementary Figure 7). These changes in signaling in 3D can lead to differential drug response in key cancer pathways [33]. Screens in 2D can both identify false lead compounds and miss identifying compounds that have a profound effects. There are a number of existing resources for drug screening bladder cell lines in 2D [15–17]; thus, the goal of this work is to contribute to the field by providing a large screening resource in 3D culture. While there have been many advances in screening bladder cancer patient-derived organoids (PDOs) [28, 29, 34], PDOs are a limited resource, and a gap remains in how to identify the best compounds from approved or IND-stage, drugs to test in screens.

In this work, we screened 652 investigational compounds and 3 clinically relevant combinations using 17 bladder cancer cell lines. The 652 compounds mostly comprise investigational new drugs or drugs that are approved for other cancer indications. The combinations tested were those most commonly used in the clinic, namely MVAC, cisplatin with gemcitabine, and carboplatin with paclitaxel [35–37]. It was notable that the DSS_3_ of the carboplatin and paclitaxel combination was superior to both MVAC and cisplatin with gemcitabine, but the maximum efficacy was lower for nearly all cell lines. These data are confirmation of what is seen in the literature, that carboplatin with paclitaxel is a preferred therapy following failure of standard of care [37].

Nearly 17% of the drugs tested showed some level of efficacy, as determined by the DSS_3_, Figure 1 [30]. Many of the compounds identified in this screen as the most efficacious have been previously implicated in bladder cancer. Romidepsin has been extensively studied in bladder cancer preclinical models, showing promise [38–41]. A Phase II clinical trial of romidepsin in bladder cancer was withdrawn (NCT00087295), tempering expectations of this drug in bladder cancer; however, recent work has shown that romidepsin spares normal cells and acts as a radiosensitizer in bladder cancer, opening the door for future studies with this drug [42]. Bortezomib has been studied in bladder cancer and failed a Stage II clinical trial, determined to be safe but not efficacious as a second line therapy [43]. Triptolide has been studied within a combination therapy in bladder pre-clinical studies [44, 45]. Though there are no current clinical trials with this drug, our results confirm previous literature that it is a promising investigational compound in bladder cancer and may warrant further studies. Many of the 30 “active” compounds (such as gemcitabine, paclitaxel, vinblastine, and doxorubicin) are currently used in the standard treatment of bladder cancer, further supporting the validity of our approach to be empirically concordant with the clinical setting. We believe that many of the remaining compounds may be worthy of further study, including screening in patient-derived organoids.

Utilizing previous sequencing efforts, we wanted to determine the impact of mutations and CNA on drug response. In particular, DNA alterations are utilized in the clinic in many basket clinical trials, such as the MATCH trial (NCT02465060) [46]. We find that bladder cell lines have a very heterogeneous mutational spectrum, with most prioritized mutations only present in 1–2 cell lines, Figure 2, consistent with current literature [47]. For mutations found in 3+ cell lines, we identify correlates with TP53 and PIK3CA mutation status. We identify onalespib and clofarabine as significantly more efficacious in TP53 mutant cell lines compared to wildtype, Supplementary Figure 1, a novel finding. Paradoxically, in the PIK3CA mutants, we identify that AZD-8186 responds best in PIK3CA WT cells, Supplementary Figure 2. This is the opposite of what we would predict clinically [48, 49]; however, it is important to note that AZD-8186 is one of many PI3K inhibitors in our drug screen, and other PI3K inhibitors with better efficacy (e.g., gedatolisib) show no significant difference in DSS_3_ between groups, supporting that PI3K mutation status alone is unlikely to be a predictor of which PI3K pathway modulating drug to use. When we incorporate PTEN mutation and CNA data, the correlation is no longer significant and we instead see a correlation with the Aurora inhibitor, MLN228, where the drug is most effective in wildtype cells, Supplementary Figure 3. It is notable that in neither the mutational nor mutational and CNA combination do we see correlations with PI3K inhibitors. Similarly, we fail to observe significant correlations of FGFR3 mutation with FGFR inhibition response, ERBB2 mutation with ERBB2 inhibition response, or HRAS and KRAS mutation and PI3K/AKT/mTOR inhibitor response. Taken together, our findings emphasize the great need for biomarkers of drug response in bladder cancer.

Integration of mutational and CNA data greatly improved our ability to identify correlations of drug response. CDKN2A loss correlated with relatively poor response to purine analogs as well as HDAC inhibitors. The purine analog result is counter to what one may expect, because CDKN2A deletion is known to increase the cell cycle, and the two drugs would be expected to have a more profound effect in highly proliferative cells [50, 51]. It is known however, that these drugs are effective in lowly proliferating lymphocytes [52, 53], and thus it is possible that they are effective in CDKN2A WT cells through a mechanism unrelated to proliferation. Previous literature has shown that some HDAC inhibitors are less efficacious in cells with TP53 loss, suggesting that different HDAC inhibitors have a differential dependence on TP53 status [54]. RB1 loss significantly correlated with average drug response across the 17 cell lines, Supplementary Figure 5. It has been established that RB1 status predicts response to cisplatin-based chemotherapy in bladder cancer [55, 56]. Our data suggest that this trend may hold for additional mechanisms of cell death.

MEK inhibition has been proposed as a promising treatment option in NMIBC by a mechanism related to enhancing the efficacy of BCG therapy [57]. Additionally, MEK inhibition has been proposed as a promising strategy in bladder cancers with high expression of KIF15, which upregulates the MEK pathway [58]. Pre-clinical work in bladder cancer [49, 59] and extensive studies in other cancers [60–63] suggest that MEK inhibition could be a potential combination approach to PI3K/AKT/mTOR inhibition, by targeting a parallel compensatory pathway. Despite these reports, the only clinical trial that utilizes MEK inhibition in bladder cancer is the ongoing MATCH screening trial in advanced solid tumors (NCT02465060), using mutation status of BRAF, GNA11, and NF1 to identify potential responders to the MEK inhibitor, Trametinib. These results suggest an opportunity to identify a population where MEK inhibition could be a particularly promising treatment strategy in bladder cancer.

Our panel of 17 cell lines have no prioritized mutations in BRAF, GNA11, or NF1, yet a subgroup responds very well to MEK inhibition. We identify the strongest responding cell lines as basal bladder cancer subtype, Figures 3 and 4. Basal bladder cancer is frequently clinically aggressive and has the worst overall prognosis [3], and thus, additional therapeutic options for these tumors would be highly clinically relevant. One cell line has a high-level EGFR amplification and another has an activating KRAS mutation, but the remaining two cell lines do not have molecular changes that would *a priori* indicate MEK sensitivity, including SCaBER, which is frequently the most sensitive cell line to MEK inhibitors. Only one other drug, atuveciclib (PTEFb/CDK9 inhibitor) (Supplementary Figure 3), was identified as significantly more effective in basal cell lines of drugs with and average DSS_3_ > 10 for basal bladder cancer, while 4 individual MEK inhibitors are significantly more effective in basal bladder cell lines, using the same criteria. Further, normalizing all MEK inhibitors with an average DSS_3_ > 0 and plotting them together, the basal subtype is significantly more sensitive to MEK inhibition than any other subtype, Figure 4C. Previous work in breast cancer has identified basal breast cancer as particularly susceptible to MEK inhibition, and importantly notes a PI3K feedback loop that requires combination inhibition to overcome the compensatory signaling [64]. Overall, this work strongly supports that MEK inhibitors should be explored as a potential therapeutic for bladder tumors with a basal signature that are refractory to standard of care therapy, particularly in combination with an inhibitor of parallel signaling.

In summary, this work is a valuable resource for the identification of experimental therapeutics in bladder cancer, having screened 652 investigational therapeutics and 3 drug combinations in 17 bladder cancer cell lines, using a 3D cell culture format. As next steps, we pose that this work be used to further test additional therapeutic options for patients with bladder cancer. Moreover, this work highlights a need for biomarkers of drug response, beyond mutational data. Lastly, using these methods, we identify MEK inhibitors as a promising therapeutic in the basal bladder cancer subtype. Important future work will investigate the specific molecular features of the basal subtype that make these cells more sensitive to MEK inhibition, and if this MEK sensitivity signature is applicable to other cancer subtypes.

## MATERIALS AND METHODS

### Cell culture

Cells used in this manuscript have all been previously published and include: 5637, UM PDX BC8149, J82, SCaBER, SW780, T-24, TCCSUP, UM-UC1, UM-UC-3, UM-UC-5, UM-UC-6, UM-UC-9, UM-UC-12, UM-UC-14, UM-UC-15, UM-UC-17, and UM-UC-18. 5637, J82, SCaBER, SW780, and TCCSUP were purchased from ATCC and expanded under ATCC recommended conditions. Remaining cell lines were expanded in HyClone DMEM w/ glutamine (Thermo Fisher Scientific), 10% HyClone FBS (Thermo Fisher Scientific), Penicillin-Streptomycin (Thermo Fisher Scientific), amphotericin b (Thermo Fisher Scientific), and gentamicin (Thermo Fisher Scientific) at 5% CO_2_. All cell lines were drug tested in 3D growth media: DMEM (Thermo Fisher Scientific), 10% FBS (Thermo Fisher Scientific), 1% matrigel (Corning), B-27 (Thermo Fisher Scientific), anti-anti (Thermo Fisher Scientific), gentamicin (Thermo Fisher Scientific), Human EGF (25 ug/500 ml) (Sigma Aldrich), Human Heregulin β-1 (25 ug/500 ml) (Stemcell Technologies), Human KGF (FGF-7)(5 ug/500 ml) (Stemcell Technologies), Human FGF-10 (5 ug/500 ml) (Stemcell Technologies), Human Noggin (50 ug/500 ml) (Stemcell Technologies), Human RSPO1 (250 ug/500 ml) (Stemcell Technologies) at 10% CO_2_.

### Chemicals and reagents

Chemicals were purchased from Selleckchem (Houston, TX, USA), Sigma-Aldrich (St. Louis, MO, USA), Cayman Chemical (Ann Arbor, MI, USA), and Med Chem Express (Monmouth Junction, NJ, USA). Included is the Selleckchem L-3500 screening library, the highly selective inhibitor library of 339 inhibitors covering 123 targets. Compounds were diluted in DMSO (Sigma-Aldrich, D2650) or water, depending on solubility, except for copanlisib which was diluted in 10% Trifluoroacetic acid in DMSO due (Sigma-Aldrich, T6508). Compounds were prepared as a 10 mM solution unless solubility constraints required lower concentrations.

### Drug screening

Cells were screened in 384-well ultra-low attachment plates (Corning, 4516 or S-Bio, MS-9384WZ) in singlicate or duplicate, 7-point dose-response format. Cells were plated on day 0 at 3000 cells per well. On day 0, drugs were added at 1:1000 using a 50 nl pin tool, resulting in 0.1% final DMSO concentration per well. On day 5, viability was measured using CellTiter-Glo 3D (Promega, G9683) on an Envision plate reader (Perkin Elmer).

### Drug sensitivity score calculation

Drug dose response data were fit to the equation Y = bottom + (top − bottom)/(1 + 10(Log10IC50-X) × HillSlope) where X = Log10 (concentration, M) and Y = % inhibition (vs. vehicle) using CDD Vault. Constraints used were bottom = 0 and top ≤ 100. DSS_3_ values were calculated as described by Yadav *et al*. [30]. IC50 value, hillslope, maximum inhibition, and drug range were entered into the DSS package for Rstudio and DSS_3_ values (ranging 0 to 100) were calculated.

### Prioritized somatic variants and copy number alterations

For all cell lines except SCaBER and TCCSUP, next-generation DNA sequencing (DNAseq) data using a targeted pan-cancer assay (Oncomine Comprehensive Panel) was previously published by our group [7, 65]. Briefly, OCP targets hotspot regions in recurrent mutated oncogenes (e.g., *BRAF* exon 15) and the entire coding region of tumor suppressor genes (e.g., *TP53*, etc.); well-supported variants with established or presumptive oncogenic roles were considered prioritized mutations. For a subset of genes with recurrent CNA, OCP targets genomic regions across the gene body to detect copy number gains and losses [65]; for the purposes of this study, only high-level amplifications (≥ 2 copy gain) or deep deletions (2 copy loss) were considered prioritized. For the SCaBER and TCCSUP cell lines, genome-wide somatic mutation and CNA data was obtained from the CCLE via cBioPortal for Cancer Genomics (http://www.cbioportal.org/) [66] and reviewed by an experienced molecular pathologist (A.M.U.); only prioritized molecular alterations (as described above) occurring within the targeted regions of OCP were included for subsequent analyses.

### RNA-based molecular subtyping

All transcriptomic data utilized in this study was generated from 2-D cultures using standard growth conditions. For all cell lines except SCaBER and TCCSUP, gene-level FPKM values were generated from whole-transcriptome next-generation RNA sequencing (RNAseq) data as described previously [31]; for the SCaBER and TCCSUP cell lines, gene-level RPKM values derived from whole-transcriptome RNAseq data were obtained directly from the CCLE database (portals. broadinstitute. org/ccle) [67]. All FPKM and RPKM values were log2-transformed, and sample-level gene expression Z-scores were generated for inter-sample standardization and comparison. For each sample, the Basal-Luminal (B-L) score was calculated as the average of basal gene expression less the average of luminal gene expression, as described previously [7, 32]. Basal genes included *CD44*, *CDH3*, *EGFR*, *TP63*, *KRT14*, *KRT16*, *KRT5*, *KRT6A*, and *KRT6B*, while luminal genes included *ERBB2*, *FGFR3*, *FOXA1*, *GATA3*, *KRT19*, *KRT20*, *PPARG*, *UPK1B*, *UPK2*, and *UPK3A*. The median B-L score across all samples was -0.15 (inter-quartile range = −0.39 to 0.55). All samples were assigned a molecular subtype based on their B-L score, as well as the average expression across all basal and luminal genes: B-L score > 0.55 was classified as “basal” subtype; B-L score < −0.39 was classified as “luminal” subtype; −0.39 ≤ B-L score ≤ 0.55 was classified as “mixed” subtype; and, average expression < 0 across all basal and luminal genes was classified as “null” subtype.

### Correlations and statistics

Significant differences in groups were first identified by using the kruskalwallis function in R to prioritize compounds. Results were then filtered to eliminate compounds that had an average DSS_3_ < 10 for all groups. Remaining significant differences were validated in GraphPad Prism 7 using either a Mann-Whitney test (2 groups) or a Dunn test (3+ groups). MEK inhibitor normalization was carried out using the scale function in R. Normalized heat maps were generated using Morpheus (Broad Institute).

## SUPPLEMENTARY MATERIALS





## Abbreviations

NMIBC: non-muscle invasive bladder cancer
MIBC: muscle invasive bladder cancer
BCG: bacille Calmette-Guerin
CCLE: Cancer Cell Line Encyclopedia
GDSC: Genomics of Drug Sensitivity in Cancer
PDO: patient-derived organoid
MVAC: methotrexate, vinblastine, adriamycin and cisplatin
DSS3: drug sensitivity score 3
HDAC: histone deacetylase
MATCH: molecular analysis for therapy choice
B-L: Basal-Luminal
