# Supplementary material for: MEK is a promising target in the basal subtype of bladder cancer
Source: Oncotarget. 2020 Nov 3;11(44):3921–32. doi: 10.18632/oncotarget.27767 (PMC7646827; doi:10.18632/oncotarget.27767)
Supplement: Supplementary file 1 [file oncotarget-11-3921-s001.pdf]

MEK is a promising target in the basal subtype of bladder cancer

SUPPLEMENTARY MATERIALS

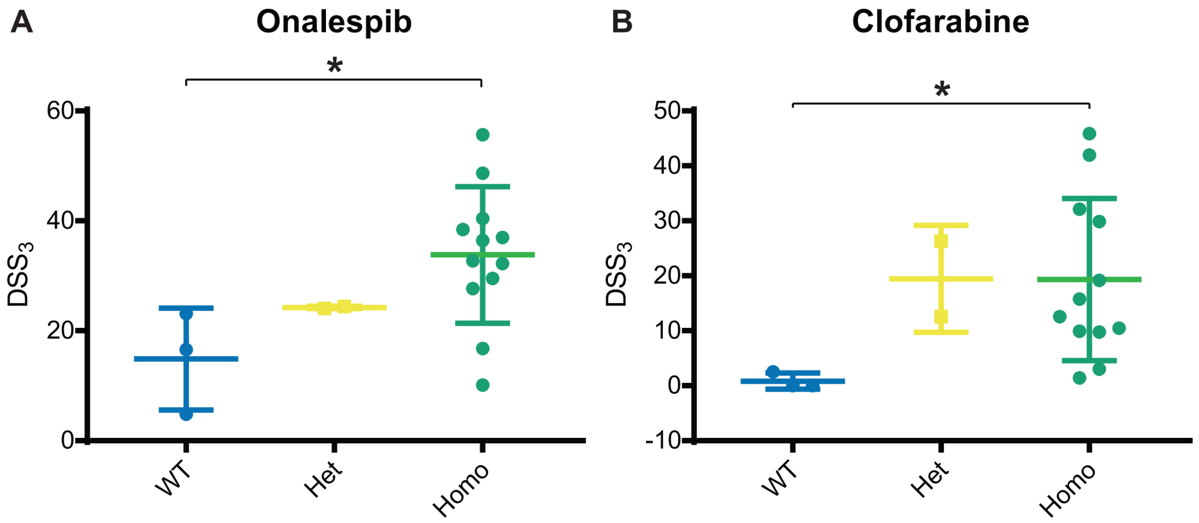

Supplementary Figure 1: Drug response correlates with TP53 mutation status. Average and standard deviation for DSS_3 response to (**A**) Onalespib and (**B**) Clofarabine, grouped by TP53 mutation status. Each point represents an individual cell line. Center line is average and brackets are standard deviation. Significance determined using Dunn test, $^{*}p < 0.05$.

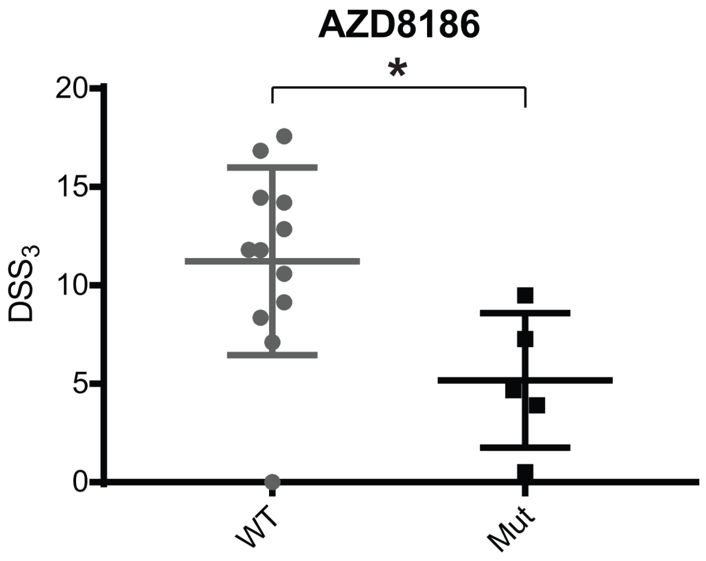

Supplementary Figure 2: AZD8186 response correlates with PIK3CA mutation status. Average and standard deviation for DSS_3 response to AZD8186, grouped by PI3K mutation status. Each point represents an individual cell line. Center line is average and brackets are standard deviation. Significance determined using Mann-Whitney test, $^{*}p < 0.05$.

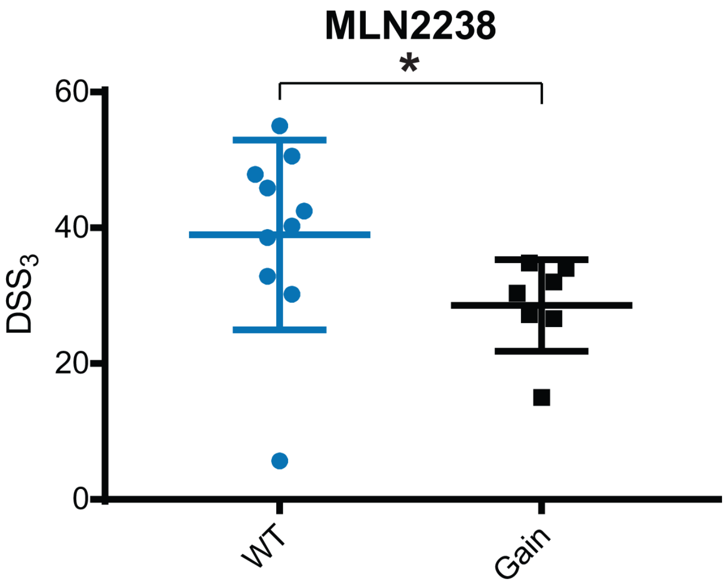

Supplementary Figure 3: MLN2238 response correlates with PI3K pathway gain. Average and standard deviation for DSS_3 response to MLN2238, grouped by PI3K mutation or PTEN deep deletion. Each point represents an individual cell line. Center line is average and brackets are standard deviation. Significance determined using Mann-Whitney test, $^{*}p < 0.05$.

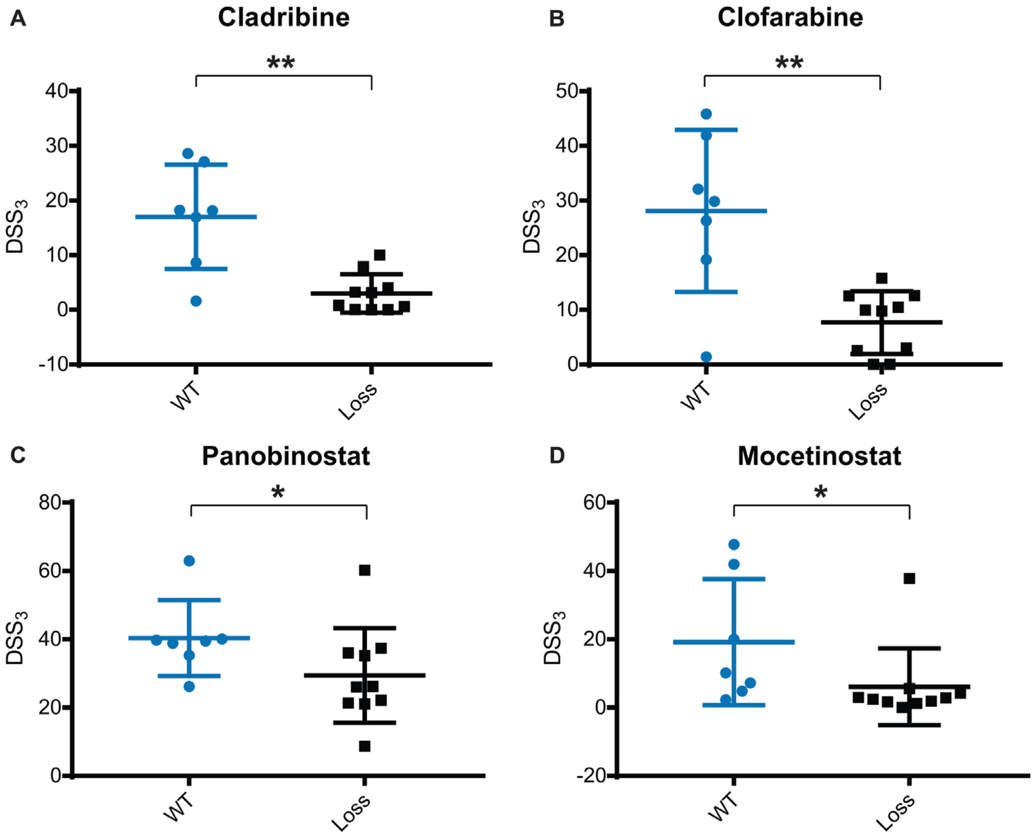

Supplementary Figure 4: Drug response correlates with CDKN2A loss. Average and standard deviation for DSS_3 response to (**A**) Cladribine, (**B**) Clofarabine, (**C**) Panobinostat, and (**D**) Mocetinostat, grouped by CDKN2A status (mutation and deep deletion combined as loss). Each point represents an individual cell line. Center line is average and brackets are standard deviation. Significance determined using Mann-Whitney test, $^{**}p < 0.01$, $^{*}p < 0.05$.

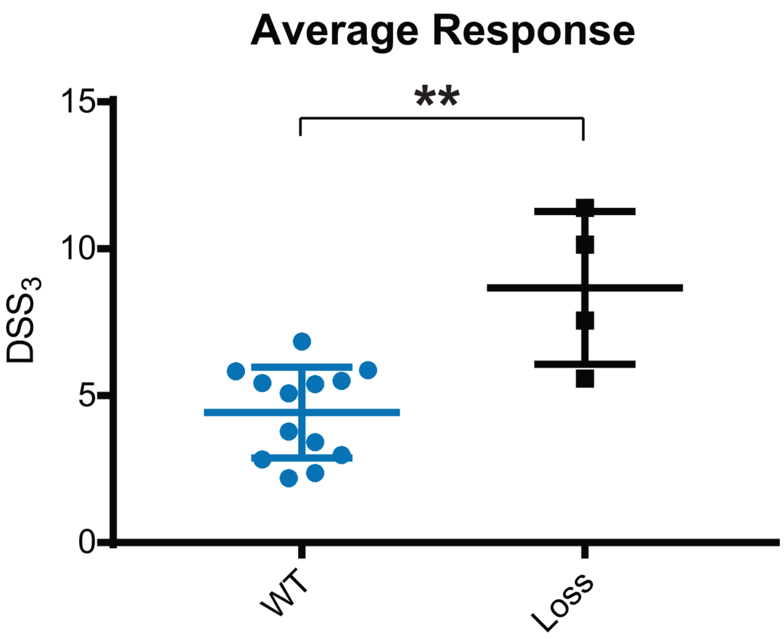

Supplementary Figure 5: Average response correlates with RB1 status. Average and standard deviation for DSS_3 average response, grouped by RB1 status (mutation and deep deletion combined as loss). Each point represents an individual cell line. Center line is average and brackets are standard deviation. Significance determined using Mann-Whitney test, $^{**}p < 0.01$.

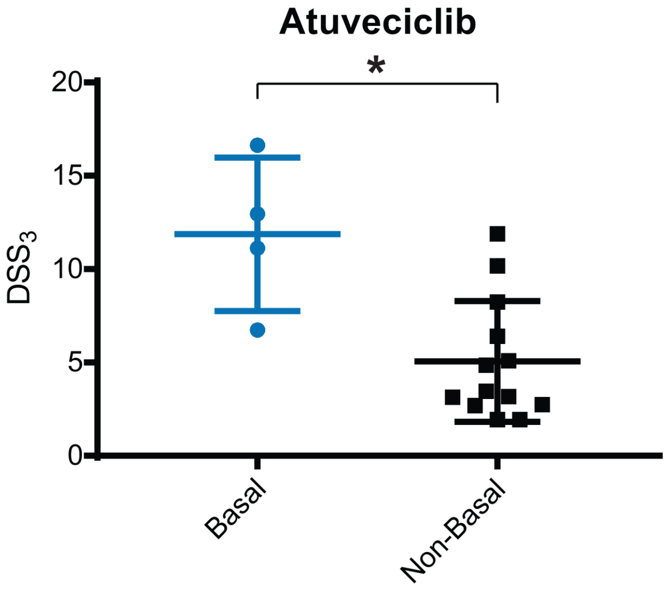

Supplementary Figure 6: Atuveciclib response correlates with basal subtype. Average and standard deviation for DSS_3 response to Atuveciclib, grouped by cell line subtype. Each point represents an individual cell line. Center line is average and brackets are standard deviation. Significance determined using Mann-Whitney test, $^{*}p < 0.05$.

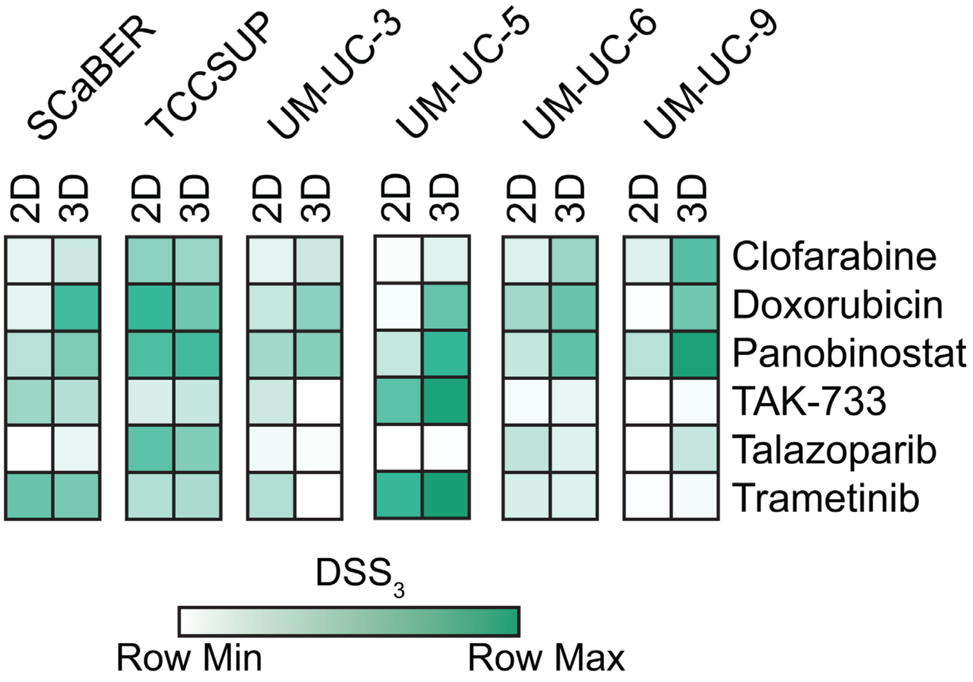

Supplementary Figure 7: Comparison of 2D and 3D screening results for prioritized compounds. Assay conditions were identical for 2D and 3D screening with the exception of adherent vs non-adherent 96-well plates. Heat map indicates DSS3, normalized by each drug.

Supplementary Table 1: DSS3 input and scores for individual cell lines and drugs. DSS3 are grouped by average drug activity classification and arranged alphabetically by drug within each activity classification. See Supplementary Table 1
